# Risk of venous thromboembolism in patients undergoing gastric cancer surgery: a systematic review and meta-analysis

**DOI:** 10.1186/s12885-023-11424-x

**Published:** 2023-10-03

**Authors:** Lin Xiang, Shuai Jin, Yang Yu, Dengfeng Wang, Hao Chen

**Affiliations:** 1 The Second Clinical Medical College, Lanzhou University, Lanzhou, China; 2 Department of Pathology, Lanzhou University Second Hospital, 82 Cuiying Gate, Lanzhou, 730030 China; 3 Department of Surgery, Qiaokou Hususu Clinic, Wuhan, China; 4 Department of Tumour Surgery, Lanzhou University Second Hospital, 82 Cuiying Gate, Lanzhou, 730030 China; 5 Department of Neurosurgery, Lanzhou University Second Hospital, Lanzhou, China; 6 The Key Laboratory of the Digestive System Tumours of Gansu Province, Lanzhou University Second Hospital, Lanzhou, China

**Keywords:** Venous thromboembolism, Incidence, Gastric cancer surgery

## Abstract

**Background:**

Venous thromboembolism (VTE) is a common postoperative complication in patients undergoing surgery for gastric cancer (GC). Although VTE incidence may vary among cancers, guidelines rarely stratify preventive methods for postoperative VTE by cancer type. The risk of VTE in patients undergoing surgery for GC remains unclear.

**Methods:**

A systematic review and meta-analysis was undertaken to determine the risk of VTE after GC surgery and discuss the clinical value of pharmacological thromboprophylaxis in these cases. Medline, Embase, Web of Science, and Cochrane Library databases were searched for articles published from their inception to September 2022.

**Results:**

Overall, 13 studies (111,936 patients) were included. The overall 1-month incidence of VTE, deep vein thrombosis (DVT), and pulmonary embolism (PE) after GC surgery was 1.8% (95% CI, 0.8–3.1%; I²=98.5%), 1.2% (95% CI, 0.5–2.1%; I²=96.1%), and 0.4% (95% CI, 0.1–1.1%; I²=96.3%), respectively. The prevalence of postoperative VTE was comparable between Asian and Western populations (1.8% vs. 1.8%; *P* > 0.05). Compared with mechanical prophylaxis alone, mechanical plus pharmacological prophylaxis was associated with a significantly lower 1-month rate of postoperative VTE and DVT (0.6% vs. 2.9% and 0.6% vs. 2.8%, respectively; all *P <* 0.05), but not PE (*P >* 0.05). The 1-month postoperative incidence of VTE was not significantly different between laparoscopic and open surgery (1.8% vs. 4.3%, *P >* 0.05).

**Conclusion:**

Patients undergoing GC surgery do not have a high risk of VTE. The incidence of VTE after GC surgery is not significantly different between Eastern and Western patients. Mechanical plus pharmacological prophylaxis is more effective than mechanical prophylaxis alone in postoperative VTE prevention. The VTE risk is comparable between open and laparoscopic surgery for GC.

**Supplementary Information:**

The online version contains supplementary material available at 10.1186/s12885-023-11424-x.

## Introduction

Gastric cancer (GC) is a common malignant tumour that is the third most common cause of cancer-related death globally [1]. Surgery is currently the primary treatment modality for resectable GC. Venous thromboembolism (VTE), including deep vein thrombosis (DVT) and pulmonary embolism (PE), is a common complication in patients with cancer. Virchow’s triad describes three elements associated with VTE, namely, blood stasis, endothelial injury, and hypercoagulability, and cancer appears to be associated with all of these elements. Cancer surgery directly damages the endothelium and activates coagulation, and postoperative patients stay in bed for a long time, resulting in blood stasis, all of which will further aggravate the risk of VTE [2–4]. The risk of VTE in cancer patients is higher than that in the general population, and major surgery is a strong risk factor for VTE [5–8]. In addition, VTE is not only a common complication of cancer surgery, but also the most common cause of death 30 days after surgery [9, 10], incurring huge medical and economic costs. Therefore, prevention of VTE in patients undergoing cancer surgery deserves more attention.

However, there is still considerable controversy regarding the development of VTE prevention strategies in patients undergoing cancer surgery, especially in different regions. The latest American Society of Clinical Oncology guideline recommends that patients undergoing surgery for major cancers should be started on thromboprophylaxis before surgery [11]. However, the American Society of Hematology only recommends postoperative thromboprophylaxis for patients undergoing surgery and considers that the evidence of its effects is still low [12]. The incidence of VTE for surgery under high-risk factors is significantly lower in Asian countries than in Western countries, and thus, the routine use of anticoagulants seems unreasonable [13, 14]. The Asian guidelines on VTE stipulate that not all cancers have a high risk of VTE. Accordingly, they only recommend drug prophylaxis for high-risk cancer surgery and mechanical prophylaxis for patients with high bleeding risk [15]. However, the guidelines do not specify which cancer surgeries are associated with a high risk of VTE. A study reported that the VTE rate is different among different races and even in the same race in different regions [16]. Various economic and medical conditions in different regions further complicate this issue, which may also be an objective factor for the differences in these guidelines. Although many studies have reported on the incidence of VTE after GC surgery, the incidence still varies widely among patients. Therefore, it is necessary to reliably evaluate the incidence of VTE after GC surgery to determine the degree of risk. Thus, this study aimed to determine the risk of VTE after GC surgery and discuss the clinical value of pharmacological thromboprophylaxis in these cases to provide a reference for the routine use of thromboprophylaxis. Towards this goal, we used a systematic review to determine the incidence of VTE in GC patients who underwent surgery.

## Methods

This review protocol is registered in the PROSPERO database (CRD42019144562) and published elsewhere [17].

### Literature search

This work has been reported in line with PRISMA (Preferred Reporting Items for Systematic Reviews and Meta-Analyses) [18] and AMSTAR (Assessing the methodological quality of systematic reviews) Guidelines [19]. We searched Medline, Embase, Web of Science, and Cochrane Library from their inception to September 2022. The keywords were as follows: thrombosis, venous thromboembolism, pulmonary embolism, PE, DVT, VTE, stomach neoplasms, stomach cancer, stomach tumour, gastric cancer, gastric tumour, epidemiologic studies, and incidence. The details of search strategy are listed in supplemental materials (Table S1).

### Inclusion and exclusion criteria

The inclusion criteria were as follows: (1) the incidence of VTE after GC surgery was reported from randomised controlled trials (RCTs), cohort studies, population-based surveys, and cross-sectional studies; (2) only studies published in English were included; (3) the type of surgery included laparoscopic or open surgery; (4) the primary outcomes included VTE events, which included symptomatic or incidentally detected DVT or PE; (5) studies should reported sufficient data to compute the VTE incidence after surgery.

The exclusion criteria were as follows: (1) patients who needed simultaneous surgery for other diseases; (2) patients with VTE at baseline; (3) studies occasionally reporting VTE as one of the adverse effects of surgery; (4) studies with no description of the lengths of follow-up.

### Study selection and data extraction

After removing duplicates, two reviewers independently screened the titles and abstracts of all articles, after which the full texts of the potentially eligible articles were retrieved and read. Any discrepancies were resolved through consultation with a third reviewer. A data extraction form was created to extract relevant information including first author, publication year, region, study design, sample size, sex, type of surgery (laparoscopic or open surgery), type of VTE (VTE, DVT, or PE) after surgery, follow-up time, thromboprophylaxis method, and bleeding complications.

### Quality assessment

Two reviewers independently assessed the quality of each included study. The bias assessment tool modified by Hoy et al. was used to assess prevalence studies [20]. Each study was evaluated based on 10 items, with each item scored as 1 (yes) or 0 (no). The risk of bias was then rated based on these scores as low (> 8), moderate (6–8), or high (≤ 5) [21]. Meanwhile, the Cochrane bias assessment tool was used for RCTs [22]. This bias assessment tool contains six domains: selection, performance, detection, attrition, reporting, and other biases. The risk of bias was classified as low, high, or unclear based on these domains.

### Statistical analysis

Study-specific incidence and SE estimates were recalculated using raw numerators and denominators from the individual studies. The metaprop procedure was used to perform a meta-analysis of proportions [23] in STATA 17.0. The procedure stabilises the variance of the study-specific incidence using the Freeman–Tukey double arcsine transformation. An overall pooled estimate of incidence was obtained using a random effects model. Heterogeneity was assessed using Cochran’s Q test and quantified using I² statistics, with I² values of 25%, 50%, and 75% representing low, medium, and high heterogeneity, respectively. Statistical significance was set at *P* < 0.05. Sensitivity analysis was performed by excluding one study at a time to observe the robustness of the results and identify the possible sources of heterogeneity. Publication bias was explored graphically using a funnel plot and statistically using the Egger’s test, and *P <* 0.05 was considered to indicate publication bias. If there was significant publication bias, the trim-and-fill method was used to solve it.

## Results

### Search results

**Fig. 1:**
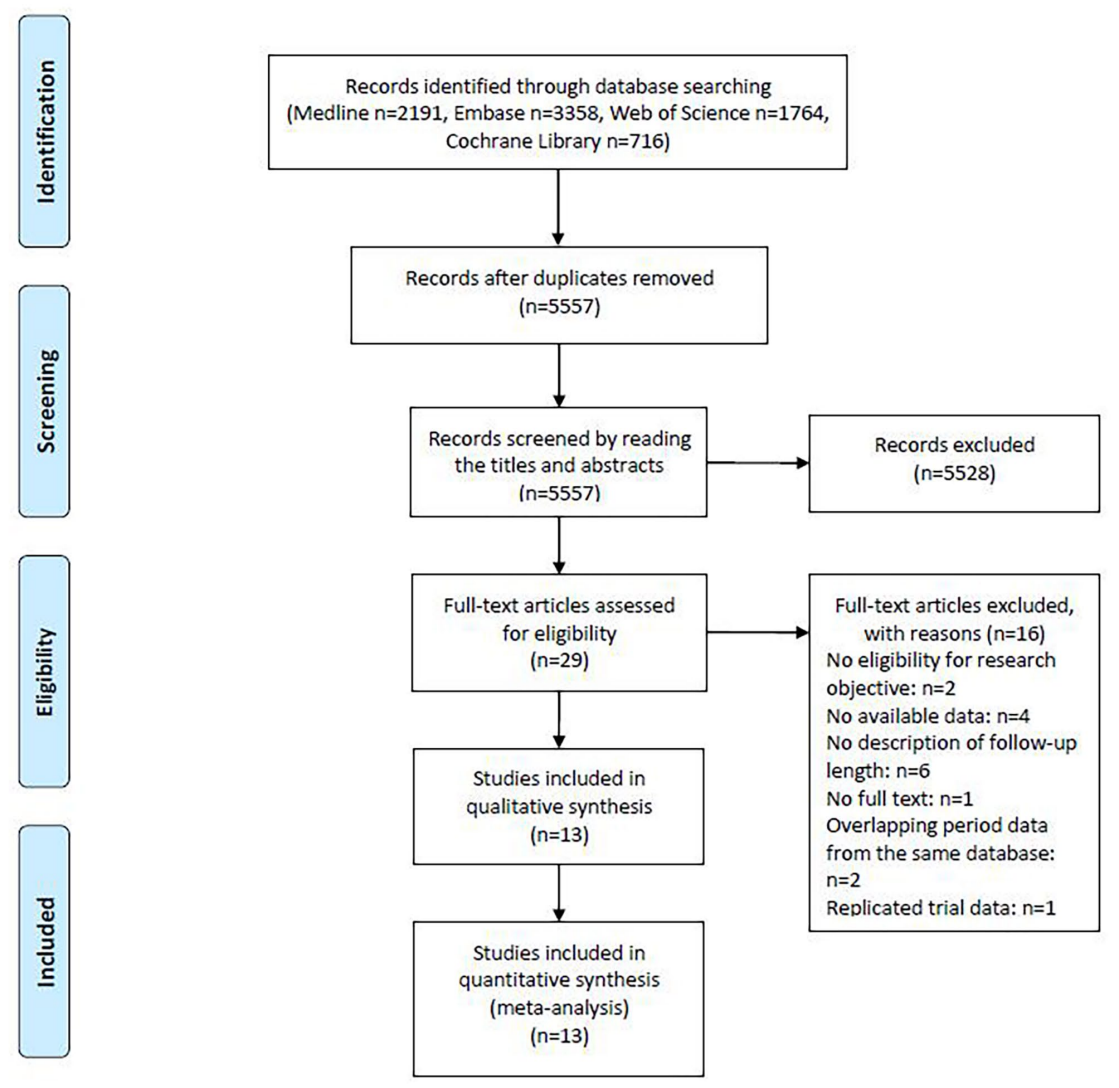
PRISMA flow chart showing article selection process

A total of 8029 records were identified during the initial literature search. After removing duplicate records, 29 eligible studies were analysed by screening titles and abstracts. The full texts of these studies were reviewed, and 16 studies were excluded for the following reasons: no eligibility for research objective, no available data, no description of follow-up length, no full text, overlapping period data from the same database, and replicated trial data. Finally, 13 studies were included in the systematic review and meta-analysis [24–36]. A flow diagram of the study selection process is shown in Fig. 1.

### Characteristics of the included studies

The characteristics of the 13 included studies are listed in Table 1. All 13 studies involved a total of 111,936 patients. The studies were conducted in Asia (n = 8, 61.5%) [26, 28–32, 34, 36], North America (n = 4, 30.8%) [25, 27, 33, 35], and Europe (n = 1, 7.7%) [24]. Western countries included England, the USA, and Canada, and Asian countries included Turkey, Korea, and Japan. In most studies, the follow-up time of VTE events after GC surgery was 1 month or similar (69.2%, n = 9) [24, 25, 27–30, 33, 35, 36]. In other studies, the patients were followed up for 7 days (15.4%, n = 2) [31, 34], 24 months (7.7%, n = 1) [32], and 36 months (7.7%, n = 1) [26].

**Table 1:** Characteristics of included studies

Study	Region	Study design	Type of data source	Sample size	Age (Mean or Median)	Gender (% Male)	Laparoscopic/ open surgery	Type of VTE	Outcome definition	Follow-up length	Prophylaxis	Bleeding complication
Adiamah 2020	England	Retrospective cohort	National healthcare database	1012	71	60.8	NR	VTE	Medical codes	30d	NR	NR
Bellini 2016	USA	Retrospective cohort	ACS-NSQIP database	3735	NR	NR	NR	VTE/DVT/PE	Medical codes	30d	NR	NR
Colapkulu-Akgul 2021	Turkey	Retrospective cohort	Single center database	27	NR	NR	NR	VTE/DVT/PE	Clinical presentation	36 m	M + P	NR
Hanna 2022	Canada	Retrospective cohort	Provincial healthcare database	3800	NR	NR	NR	VTE	Medical codes	30d	NR	NR
Jung 2018	Korea	RCT	Clinical trial	666	57.4 ± 10/ 57.9 ± 10.9	65.3	411/255	VTE/DVT/PE	Routine DUS	30d	M / M + P	Recorded
Kaida 2021	Japan	Prospective cohort	Observational study	126	NR	75.4	60/66	VTE	Clinical presentation	30d	M + P (some of patients)	NR
Kim 2013	Korea	Prospective cohort	Observational study	375	61	67.5	279/96	VTE/DVT/PE	Routine DUS	4w	M	NR
Kimura 2016	Japan	Prospective cohort	Observational study	36	NR	NR	36/0	VTE/DVT/PE	Routine DUS	7d	M + P (some of patients)	NR
Lee 2010	Korea	Prospective cohort	Single center database	1627	NR	NR	NR	VTE	Clinical presentation	24 m	NR	NR
Mallick 2022	USA	Retrospective cohort	HCUP Nationwide Readmissions Database	4586	NR	NR	NR	VTE/DVT/PE	Medical codes	30d/90d	NR	NR
Osaki 2018	Japan	Retrospective cohort	Observational study	153	NR	NR	NR	VTE/DVT/PE	Routine DUS	7d	M / P / M + P	NR
Ruff 2019	USA	Retrospective cohort	ACS-NSQIP database	8384	66	62.4	NR	VTE	Clinical presentation	30d	NR	NR
Yhim 2014	Korea	Retrospective cohort	The Korean HIRA database	87,409	NR	NR	NR	VTE/DVT/PE	Medical codes	5w	NR	NR

NR, not reported; DVT, deep vein thrombosis; PE, pulmonary embolism; M, mechanical; P, pharmacological; DUS, duplex ultrasonography; ACS-NSQIP, The American College of Surgeons National Surgical Quality Improvement Program; HCUP, Healthcare Cost and Utilization Project; HIRA, Health Insurance Review and Assessment Service

Of the 13 studies included, there were 12 prevalence studies and 1 RCT. In total, 4 and 9 of the studies had a low and moderate risk of bias, respectively (Table S2, Fig. S1).

### Incidence of VTE, DVT, and PE after GC surgery

**Fig. 2:**
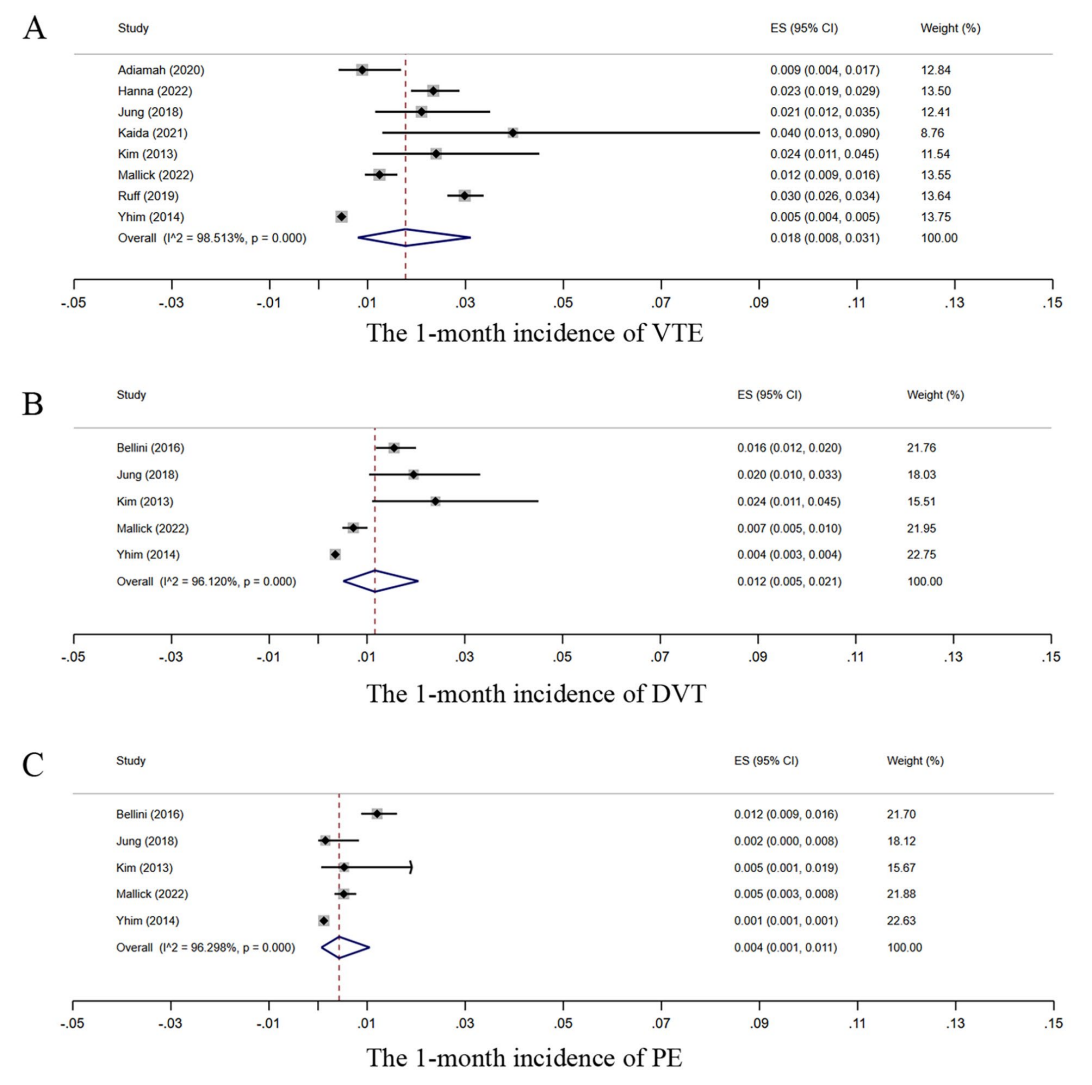
Forest plots of postoperative VTE, DVT, and PE within 1 month in GC

Nine studies [24, 25, 27–30, 33, 35, 36] with a 1-month follow-up reported the incidence of postoperative VTE in patients undergoing GC surgery. As some studies used data from the same database (ACS-NSQIP), the study by Ruff 2013 [35] with the largest cohort was retained for the meta-analysis, while the study by Bellini 2016 [25] with duplicate analyses was excluded. The overall 1-month incidence of VTE after GC surgery was 1.8% (95% CI, 0.8–3.1%; I²=98.5%) (Fig. 2A). Five studies [25, 28, 30, 33, 36] with a follow-up period of 1 month reported postoperative DVT events. The overall 1-month incidence of DVT after GC surgery was 1.2% (95% CI, 0.5–2.1%; I²=96.1%) (Fig. 2B). Five studies [25, 28, 30, 33, 36] documented PE events within 1 month, and the 1-month incidence of postoperative PE was 0.4% (95% CI, 0.1–1.1%; I²=96.3%) (Fig. 2C). There were high levels of heterogeneity for all the above results. Further sensitivity analyses showed that there was no significant change in the results and heterogeneity level, indicating that these results were stable and reliable (Fig. S2).

**Fig. 3:**
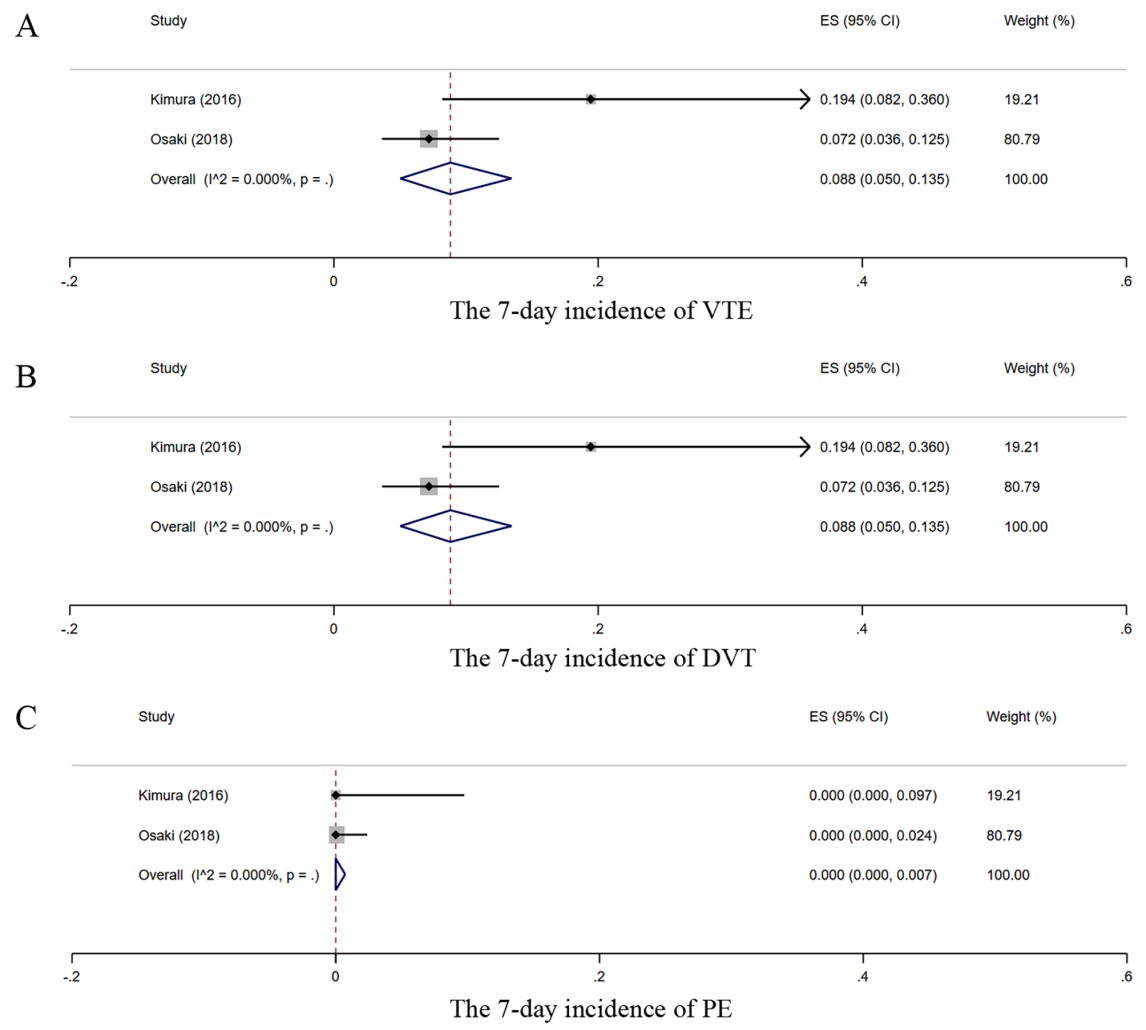
Forest plots of postoperative VTE, DVT, and PE within 7 days in GC

Two studies [31, 34] assessed VTE events within 7 days after GC surgery. The 7-day incidence of VTE, DVT, and PE was 8.8% (95% CI, 5.0–13.5%; I²=0%), 8.8% (95% CI, 5.0–13.5%; I²=0%), and 0% (95% CI, 0–0.7%; I²=0%), respectively (Fig. 3). Three studies [26, 32, 33] reported VTE events within 90 days, 24 months, and 36 months after GC surgery. The study by Lee 2010 [32] with a follow-up period of 24 months reported only the total VTE events and did not specify DVT and PE. The incidence rates of postoperative VTE at 90 days, 24 months, and 36 months were 2.4%, 2.0%, and 3.7%, respectively. The incidence rates of DVT at 90 days and 36 months postoperatively were 1.4% and 3.7%, respectively. The incidence rates of PE at 90 days and 36 months postoperatively were 1.0% and 0%, respectively.

### Subgroup analysis

Studies with a 1-month follow-up after GC surgery [24, 25, 27–30, 33, 35, 36] were selected for subgroup analysis. With respect to region, the 1-month incidence of VTE, DVT, and PE in patients undergoing GC surgery was 1.8% (95% CI, 1.0–2.8%; I²=94.7%), 1.1% (95% CI, 0.8–1.3%), and 0.8% (95% CI, 0.6–1.0%) in non-Asian regions, respectively and was 1.8% (95% CI, 0.4–4.0%; I²=92.3%), 1.3% (95% CI, 0.1–3.5%), and 0.1% (95% CI, 0–0.4%) in Asian regions, respectively. There was no significant difference in the 1-month incidence of postoperative VTE and DVT between non-Asian and Asian GC patients (*P >* 0.05); however, the 1-month incidence of PE after GC surgery in Asian regions was significantly lower than that in non-Asian regions (0.1% vs. 0.8%, *P <* 0.01) (Table 2).

**Table 2:** Subgroup analysis of the 1-month incidence of VTE and subgroup differences

Subgroups	VTE				DVT				PE		
	Number of studies	Incidence % (95% CI)	*P*_heterogeneity_		Number of studies	Incidence % (95% CI)	*P*_heterogeneity_		Number of studies	Incidence % (95% CI)	*P*_heterogeneity_
**Region**											
Non-Asia	4	1.8 (1.0–2.8)	0.920		2	1.1 (0.8–1.3)	0.704		2	0.8 (0.6–1.0)	0.000
Asia	4	1.8 (0.4–4.0)			3	1.3 (0.1–3.5)			3	0.1 (0.0–0.4)	
**Study design**											
Retrospective	5	1.4 (0.5–3.0)	0.222		3	0.8 (0.2–1.6)	0.018		3	0.5 (0.1–1.4)	0.646
Prospective	3	2.3 (1.5–3.3)			2	2.1 (1.3–3.1)			2	0.3 (0.0–0.7)	
**Diagnostic method**											
Non-routine DUS					3	0.8 (0.2–1.6)	0.018				
Routine DUS					2	2.1 (1.3–3.1)					
**Prophylaxis method**											
Mechanical	2	2.9 (1.8–4.3)	0.007		2	2.8 (1.7–4.2)	0.010		2	0.4 (0.0–1.1)	0.163
Mechanical + Pharmacological	1	0.6 (0.1–2.2)			1	0.6 (0.1–2.2)			1	0.0 (0.0–1.1)	
**Surgery type**											
Open	2	4.3 (1.5–8.2)	0.138								
Laparoscopic	2	1.8 (0.5–3.7)									

With respect to the methods used to identify VTE cases, 2 of the 9 studies with a 1-month follow-up used routine postoperative imaging screening [28, 30]. Considering that the routine imaging tool in the 2 studies was duplex ultrasonography (DUS), we set the target for analysis as DVT. The 1-month incidence of postoperative DVT was significantly lower in the non-routine imaging group than in the routine imaging group (0.8% (95% CI, 0.2–1.6%) vs. 2.1% (95% CI, 1.3–3.1%), *P* < 0.05; Table 2).

Four studies clearly described the thromboprophylaxis methods, including 1 clinical trial [28] and 1 observational [30] study all with a 1-month follow-up, 1 observational study with a 7-day follow-up [34], and 1 database analysis study with a 36-month follow-up [26]. The 1-month incidence of postoperative VTE, DVT, and PE was 2.9% (95% CI, 1.8–4.3%), 2.8% (95% CI, 1.7–4.2%), and 0.4% (95% CI, 0–1.1%), respectively, in patients receiving mechanical prophylaxis alone. Meanwhile, it was 0.6% (95% CI, 0.1–2.2%), 0.6% (95% CI, 0.1–2.2%), and 0% (95% CI, 0–1.1%), respectively, in patients receiving mechanical prophylaxis plus anticoagulant. Although the incidence rates of VTE, DVT, and PE were lower with combined anticoagulants than with mechanical prophylaxis alone, only the incidence rates of VTE and DVT were significantly different (0.6% vs. 2.9% and 0.6% vs. 2.8%, respectively; all *P <* 0.05) (Table 2). The study with a 7-day follow-up [34] also reported VTE rates of 7.5%, 0%, and 0% in the mechanical prophylaxis alone group, pharmacological prophylaxis alone group, and mechanical plus pharmacological prophylaxis group, respectively. Additionally, in the study with a 36-month follow-up [26], the incidence of VTE, DVT, and PE was 3.7%, 3.7%, and 0%, respectively, in patients receiving mechanical plus pharmacological prophylaxis. Among the above-mentioned 4 studies, only 1 study [28] reported postoperative bleeding complications. The 1-month bleeding rate was significantly lower in patients with mechanical thromboprophylaxis than in those with mechanical plus pharmacological prophylaxis (1.2% vs. 9.1%, *P <* 0.01) [28].

Two studies [29, 30] reported the 1-month incidence of VTE according to the type of surgery. The incidence of VTE in patients after open surgery was 4.3% (95% CI, 1.5–8.2%) and was 1.8% (95% CI, 0.5–3.7%) after laparoscopic surgery. Despite the higher incidence in open surgery than in laparoscopic surgery, the difference was not statistically significant (*P >* 0.05) (Table 2).

Forest plots for all subgroup analyses were presented in supplemental materials (Fig. S3-5).

### Publication bias

Exploration of publication bias found that there was only significant publication bias in studies of the 1-month postoperative DVT in GC (Egger’s test, *P* < 0.05) (Fig. S6). Next, the bias was solved by the trim-and-fill method, and 3 studies were virtualised. However, the final outcome did not change, and the results of the existing meta-analysis were considered to be stable (Fig. S7).

## Discussion

Despite the varying incidence of VTE among cancers, current guidelines on preventive methods are not well stratified by cancer type. This study found that the overall 1-month incidence rate of VTE, DVT, and PE after GC surgery was low. These results are consistent with those of a previous meta-analysis of postoperative symptomatic VTE in abdominal and pelvic tumours [37]. However, we only focused on GC and did not limit VTE presentation (symptomatic or asymptomatic). Most current guidelines do not describe VTE prevention after GC surgery, and recommendations for VTE prophylaxis are not specified according to the types of cancer and surgery [11, 12, 15, 38, 39]. Considering that the reported incidence of postoperative VTE varies widely among pelvic and abdominal tumours (e.g., 1.9% for colorectal cancer and as high as 8% for ovarian cancer) [40, 41], it is necessary to understand the accurate incidence of this complication in patients undergoing GC surgery.

Although the 1-month incidence of VTE (1.8%) and DVT (1.2%) after GC surgery is low, the 7-day incidence of postoperative VTE (8.8%) and DVT (8.8%) is high. Such a high rate of VTE may be due to the routine postoperative imaging screening in both studies with a 7-day follow-up; all patients diagnosed with VTE were asymptomatic [31, 34]. Although routine imaging screening can reveal asymptomatic VTE, it is not recommended because of the cost and limited accuracy of imaging examination in the diagnosis of asymptomatic VTE [42]. Asymptomatic VTE events are very common in cancer patients, and the associated mortality is not lower than that of symptomatic VTE events [43–45]. In the current study, analysis stratified according to VTE diagnostic methods showed that the 1-month incidence of postoperative DVT was significantly higher in the routine DUS group than in the non-routine DUS group, which indicated that the incidence of asymptomatic DVT was higher than that of symptomatic DVT. Considering that PE is mainly a complication of DVT, and PE accompanying DVT accounts for 80.6% of the total incidence of PE [46], clinicians have to pay more attention to PE due to its potential lethality. At present, routine imaging screening is rarely performed in the clinic and is only used to confirm the diagnosis when patients have relevant clinical manifestations, which may mean that many asymptomatic VTEs are missed. Therefore, whether postoperative imaging screening should be routinely performed remains controversial.

Many previous studies have shown that the incidence of VTE in Asian populations is significantly lower than that in Western populations [47–52]. Surprisingly, in our study, the pooled 1-month incidence of VTE after GC surgery was similar between Asian and Western countries. In addition, although the rate of postoperative DVT was higher in Asia than in the West, the difference was not statistically significant. Meanwhile, the incidence of PE after GC surgery was significantly lower in Asian countries than in Western countries. Sakon et al. reported that the PE rate after general surgery was lower in Japan than in the West (0.33% vs. 1.6%) [53], and consistent findings of a difference between Asian and Western populations were found in the current study. Currently guidelines, Western guidelines put more emphasis on pharmacological thromboprophylaxis, while Asian guidelines do not. Our study found that the 1-month incidence of VTE in patients undergoing GC surgery in both Asian and Western populations was not high at 1.8%, which suggested that there may be futile treatment in the Western guidelines. However, among the included Western studies, none reported specific methods for VTE prevention. Considering that drug prophylaxis in patients undergoing cancer surgery is used routinely in Western countries but rarely in Asian countries, this consistency between the VTE rates in the East and West may only be a facade. Therefore, this result should be interpreted cautiously, and more complete data need to be obtained for further analyses.

The appropriate strategy for pharmacological thromboprophylaxis in patients undergoing cancer surgery also remains unclear to date. Anticoagulant use for patients undergoing cancer surgery is routine practice in Western countries, and the debate is mostly focused on drug selection and the duration of prophylaxis. The latest ITAC and ESMO guidelines recommended the highest prophylactic dose of LMWH once per day for patients undergoing major cancer surgery, starting 2–12 h preoperatively and extending to 4 weeks after surgery [54, 55]. In the guidelines from the Japanese Circulation Society, patients over the age of 40 undergoing major cancer surgery were considered to be at high risk of VTE, and anticoagulation therapy was recommended as a preventive treatment for patients undergoing abdominal surgery [56]. The choice of anticoagulants includes enoxaparin, fondaparinux, or low-dose unfractionated heparin. The medication regimen was as follows: starting 24 h postoperatively, enoxaparin was administered subcutaneously twice daily at a dose of 2000U with a treatment duration of ≤ 2 weeks, or fondaparinux subcutaneously once daily at a dose of 2.5 mg with a treatment duration of ≤ 8 days. However, in Korean guidelines for patients undergoing GC surgery, patients aged < 60 years were defined to have very low risk, and only early ambulation was recommended; meanwhile patients aged ≥ 60 years were defined to have low risk, and mechanical prevention was recommended [57]. The latest Asian venous thromboembolism guidelines recommend LMWH for VTE prevention in patients undergoing cancer surgery, but lack details such as drug dosage [15]. But in fact, most doctors in Japan and Korea still prefer mechanical prophylaxis over pharmacological prophylaxis. Currently, guidelines from the Asian region are still fewer and slower to update compared with Western countries. In the final analysis, the crux of the controversy is postoperative bleeding, the most common side effect of pharmacological prophylaxis in cancer surgery. A previous RCT showed that mechanical prophylaxis combined with fondaparinux was associated with significantly lower incidence of postoperative VTE than was mechanical prophylaxis alone in patients undergoing abdominal surgery, but there was more severe postoperative bleeding [58]. Similarly, another study on abdominal tumour surgery also reported that although the 1-month incidence of postoperative VTE in patients who received in-hospital drug prevention before and after surgery was as low as 0.35%, up to 42% of the patients had major bleeding [59]. In patients undergoing GC surgery, Joe et al. found that although the use of LMWH brought significant benefits, it also significantly increased the risk of bleeding [60]. In our study, mechanical prophylaxis combined with anticoagulants was associated with a significantly lower risk of VTE within 1 month after GC surgery than was mechanical thromboprophylaxis alone. However, postoperative bleeding events were also significantly higher in the study by Jung 2018 [28]. Interestingly, all the included studies comparing between mechanical prophylaxis alone and mechanical plus pharmacological prophylaxis were from Japan and Korea. This may reflect the scepticism of Asian surgeons about the benefits of chemoprophylaxis. In addition, only one of the included studies reported postoperative bleeding events with different preventive measures, so more studies are needed to provide reliable data.

Only few studies have focused on the long-term VTE risk after GC surgery, and most related studies have focused on VTE events within 1 month. An earlier prospective cohort study of national populations found that although the VTE risk peaked at approximately 3 weeks postoperative, it is still high at 12 weeks postoperative [61]. In addition, in two analyses of the national readmission database, the readmission rate related to VTE after GC surgery was remained high over a long period [33, 62]. In the current analysis, only 3 of the 13 studies reported the long-term risk of VTE after GC surgery. Despite the limited number of included studies, it still showed an increasing trend in the rate of VTE from the time after GC surgery: 1 m, 1.8%; 90d, 2.4%; 24 m, 2.0%; 36 m, 3.7% for VTE; 1 m, 1.2%, 90d, 1.4%; and 36 m, 3.7% for DVT; and 1 m, 0.4%; 90d, 1.0%, and 36 m, 0% for PE. However, the association between GC surgery and long-term VTE risk is still unconvincing, because the long-term risk may be due to other causes, such as tumour recurrence, rather than the cancer surgery itself.

Laparoscopic surgery has become widely used in cancer surgery; however, although this surgical approach has the advantages of minimal trauma and rapid postoperative recovery, its impact on VTE is still controversial. One study suggested that the relatively longer duration of laparoscopic surgery and the compression caused by the pneumoperitoneum might increase postoperative VTE events [63]. However, some studies found that the risk of VTE after laparoscopic surgery was comparable to that after open surgery [34, 59]. The results of two studies using large clinical databases supported that laparoscopic surgery has a significantly lower incidence of postoperative VTE than has open surgery [64, 65]. In the current analysis, the incidence of postoperative VTE was higher after open surgery than after laparoscopic surgery, but the difference was not statistically significant. Few studies have compared VTE risk after different surgical procedures for GC, and thus, more studies are needed to further evaluate this risk in the future.

Among the 13 studies included in the current analysis, 6 studies involved 5 national or provincial databases. The incidence of postoperative VTE after GC surgery was 0.89–2.98% from Western databases [24, 25, 27, 33, 35]. Meanwhile, the incidence of VTE reported from only one Asian database was 0.47% [36]. Although some of the data reported from these studies, such as those on thromboprophylaxis methods, are incomplete, they are still of great reference for real-world data. Eastern and Western countries showed significant differences in both the main sites of GC and the treatment methods. Proximal GC is common in Western populations, whereas distal GC is more prevalent in Asia [66]. In addition, gastrectomy with D1 lymphadenectomy is mostly used in the West, and attention is paid to perioperative chemoradiation; meanwhile, D2 lymphadenectomy is most commonly performed for GC in Asian countries [67, 68]. Chemoradiation is an important risk factor for postoperative VTE in cancer [69, 70], and this may be one of the reasons for the clinical use of drug prophylaxis in the West. In contrast, the more aggressive surgical procedures for GC in Asia than in Western countries may explain why Asian surgeons are more worried about postoperative bleeding caused by anticoagulants. Therefore, these differences may have contributed to the disagreement between the East and West on pharmacological thromboprophylaxis for patients undergoing GC surgery.

Our study determined the postoperative incidence of VTE in patients undergoing GC surgery, providing a reference for clinicians to formulate preventive measures. This meta-analysis has several strengths. First, the analyses only considered studies whose primary outcomes included VTE events to ensure reliability of the results. Second, the studies were required to include a clear follow-up period, and the VTE data were classified and analysed according to similar lengths of follow-up. Third, the risk of postoperative VTE and bleeding complications with pharmacological thromboprophylaxis was evaluated, although this was observed only in Asian populations.

Despite its strengths, this study also has some limitations. There was substantial heterogeneity among the included studies, which might have resulted from the different methods used to identify VTE, lack of information about prevention methods, and unknown dosage of LMWH, etc. In addition, the included studies were only from six countries, and most of the studies were from the USA, Japan, and Korea, which may affect the representativeness of the estimates. Therefore, further research is needed in other Eastern and Western countries. Moreover, as none of the included studies from Western countries described the use of anticoagulation and postoperative bleeding complications, the benefit of chemoprophylaxis in Western populations could not be assessed. More prospective studies are needed to optimise the risk stratification of patients and develop more personalised treatment plans to achieve the optimal balance between the risk of VTE and bleeding prevention. Finally, because the demographic information provided by most of the included studies only reported pooled data for multiple tumours (e.g. esophagogastric, gastrointestinal tract, and pelvic/abdominal cancers) and did not provide biological data for GC alone, there were insufficient data to further investigate the biological factors underlying the differences in VTE incidence between Asian and Western populations. Therefore, more detailed data are needed to elucidate the biological factors that may cause the difference in VTE rates.

In conclusion, the 1-month incidence of VTE in patients undergoing GC surgery is not very high. The incidence rate of VTE after GC surgery is comparable between Eastern and Western patients; however, evidence, such as for thromboprophylaxis measures, in different regions is still lacking. Additionally, pharmacological prophylaxis significantly increases the risk of postoperative bleeding complications after GC surgery in Asian populations, although VTE events are also notably reduced. Compared with open GC surgery, laparoscopic GC surgery does not increase or reduce the risk of postoperative VTE.

### Electronic supplementary material

Below is the link to the electronic supplementary material.

Supplementary Material 1

## Data Availability

The data in this study are available from the article and its supplementary materials.
