# Supplementary material for: Risk of venous thromboembolism in patients undergoing gastric cancer surgery: a systematic review and meta-analysis
Source: BMC Cancer. 2023 Oct 3;23:933. doi: 10.1186/s12885-023-11424-x (PMC10546706; doi:10.1186/s12885-023-11424-x)
Supplement: Supplementary file 1 — Supplementary Material 1 [file 12885_2023_11424_MOESM1_ESM.docx]

**Supplemental materials**

**Table S1 Search strategy**

**Table S2 Risk of bias for prevalence studies**

**Fig. S1 Risk of bias for RCTs**

**Fig. S2 Sensitivity analysis. (A) 1-month incidence of VTE, (B) 1-month incidence of DVT, (C) 1-month incidence of DVT**

**Fig. S3 Forest plots of subgroup analysis for postoperative VTE within 1 month**

**Fig. S4 Forest plots of subgroup analysis for postoperative DVT within 1 month**

**Fig. S5 Forest plots of subgroup analysis for postoperative PE within 1 month**

**Fig. S6 Funnel plots for the publication bias. (A) postoperative VTE within 1 month, (B) postoperative DVT within 1 month, (C) postoperative PE within 1 month**

**Fig. S7 The trim-and-fill method for the publication bias of postoperative DVT within 1 month**

**Table S1 Search strategy**

| 1 | "Thrombosis"[Mesh] OR "Thromboembolism"[Mesh] OR "Venous Thromboembolism"[Mesh] OR "Venous Thrombosis"[Mesh] OR "Pulmonary Embolism"[Mesh] |
| --- | --- |
| 2 | "thrombus*"[Title/Abstract] OR "thrombotic*"[Title/Abstract] OR "thrombolic*"[Title/Abstract] OR "thromboemboli*"[Title/Abstract] OR "thrombos*"[Title/Abstract] OR "embol*"[Title/Abstract] |
| 3 | "PE"[Title/Abstract] OR "DVT"[Title/Abstract] OR "VTE"[Title/Abstract] |
| 4 | ("vein*"[Title/Abstract] OR "venous"[Title/Abstract]) AND "thromb*"[Title/Abstract] |
| 5 | #1-4 OR |
| 6 | "carcin*"[Title/Abstract] OR "cancer*"[Title/Abstract] OR "neoplas*"[Title/Abstract] OR "tumour*"[Title/Abstract] OR "tumor*"[Title/Abstract] OR "cyst*"[Title/Abstract] OR "growth*"[Title/Abstract] OR "adenocarcin*"[Title/Abstract] OR "malig*"[Title/Abstract] |
| 7 | "intestin*"[Title/Abstract] OR "digest*"[Title/Abstract] OR "gastr*"[Title/Abstract] OR "gut"[Title/Abstract] OR "epigastr*"[Title/Abstract] OR "stomach*"[Title/Abstract] |
| 8 | #6 AND #7 |
| 9 | "Abdominal Neoplasms"[Mesh] OR "Intestinal Neoplasms"[Mesh] OR "Stomach Neoplasms"[Mesh] |
| 10 | #8 OR #9 |
| 11 | "Epidemiologic Studies"[Mesh] OR "Case-Control Studies"[Mesh] OR "Cohort Studies"[Mesh] OR "Incidence"[Mesh] |
| 12 | "case control"[Title/Abstract] OR "cohort study"[Title/Abstract] OR "cohort studies"[Title/Abstract] OR "cohort analy*"[Title/Abstract] OR "follow up study"[Title/Abstract] OR "follow up studies"[Title/Abstract] OR "observational study"[Title/Abstract] OR "observational studies"[Title/Abstract] OR "longitudinal"[Title/Abstract] OR "retrospective"[Title/Abstract] |
| 13 | #11 OR #12 |
| 14 | #5 AND #10 AND #13 |

**Table S2 Risk of bias for prevalence studies**

| Study | Q1 | Q2 | Q3 | Q4 | Q5 | Q6 | Q7 | Q8 | Q9 | Q10 | Total score | Overall risk Assessment |
| --- | --- | --- | --- | --- | --- | --- | --- | --- | --- | --- | --- | --- |
| Adiamah 2020 | Y | Y | Y | Y | Y | Y | N | Y | Y | Y | 9 | Low |
| Bellini 2016 | N | N | Y | Y | Y | Y | Y | Y | Y | Y | 8 | Moderate |
| Colapkulu-Akgul 2021 | N | N | Y | N | Y | Y | Y | Y | N | Y | 6 | Moderate |
| Hanna 2022 | N | Y | Y | Y | Y | Y | N | Y | Y | Y | 8 | Moderate |
| Kaida 2021 | N | N | Y | Y | Y | Y | Y | Y | Y | Y | 8 | Moderate |
| Kim 2013 | N | N | Y | Y | Y | Y | Y | Y | Y | Y | 8 | Moderate |
| Kimura 2016 | N | N | N | N | Y | Y | Y | Y | Y | Y | 6 | Moderate |
| Lee 2010 | N | N | Y | Y | Y | Y | Y | Y | N | Y | 7 | Moderate |
| Mallick 2022 | Y | Y | Y | Y | Y | Y | N | Y | Y | Y | 9 | Low |
| Osaki 2018 | N | N | Y | Y | Y | Y | Y | Y | Y | Y | 8 | Moderate |
| Ruff 2019 | N | N | Y | Y | Y | Y | Y | Y | Y | Y | 8 | Moderate |
| Yhim 2014 | Y | Y | Y | Y | Y | Y | N | Y | Y | Y | 9 | Low |

1. Was the study’s target population a close representation of the national population in relation to relevant variables, e.g., age, sex, occupation?
2. Was the sampling frame a true or close representation of the target population?
3. Was some form of random selection used to select the sample, OR, was a census undertaken?
4. Was the likelihood of non-response bias minimal?
5. Were data collected directly from the subjects (as opposed to a proxy)?
6. Was an acceptable case definition used in the study?
7. Was the study instrument that measured the parameter of interest (e.g.prevalence of low back pain) shown to have reliability and validity (if necessary)?
8. Was the same mode of data collection used for all subjects?
9. Was the length of the shortest prevalence period for the parameter of interest appropriate?
10. Were the numerator(s) and denominator(s) for the parameter of interest appropriate?
11. Summary item on the overall risk of study bias.

**Fig. S1 Risk of bias for RCTs**


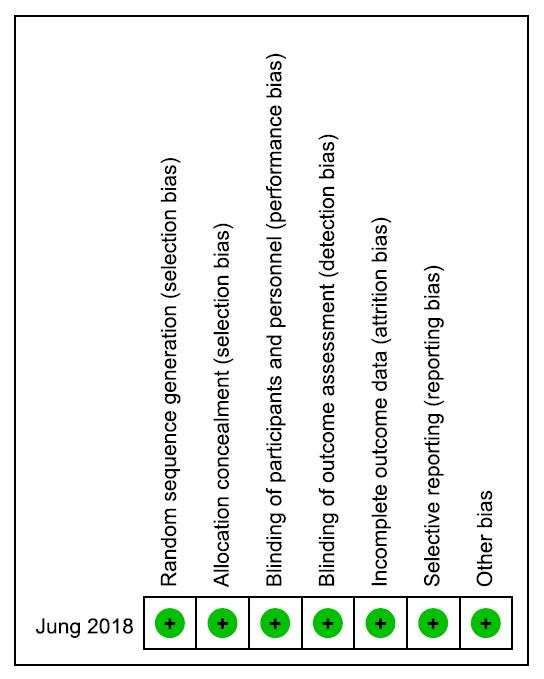

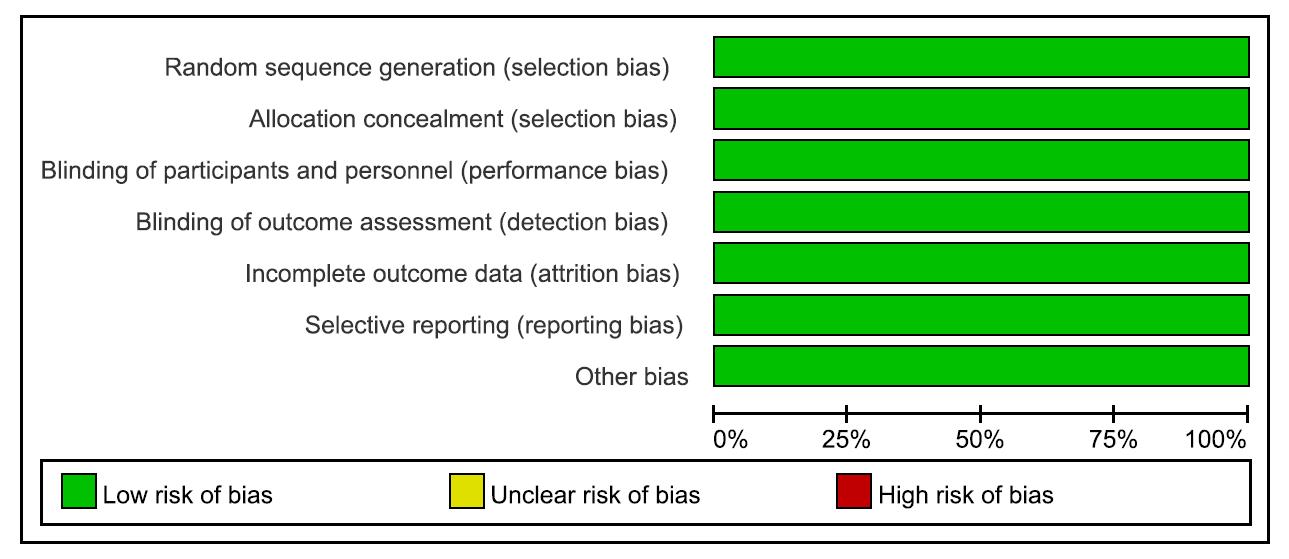


**Fig. S2 Sensitivity analysis. (A) 1-month incidence of VTE, (B) 1-month incidence of DVT, (C) 1-month incidence of DVT**

**
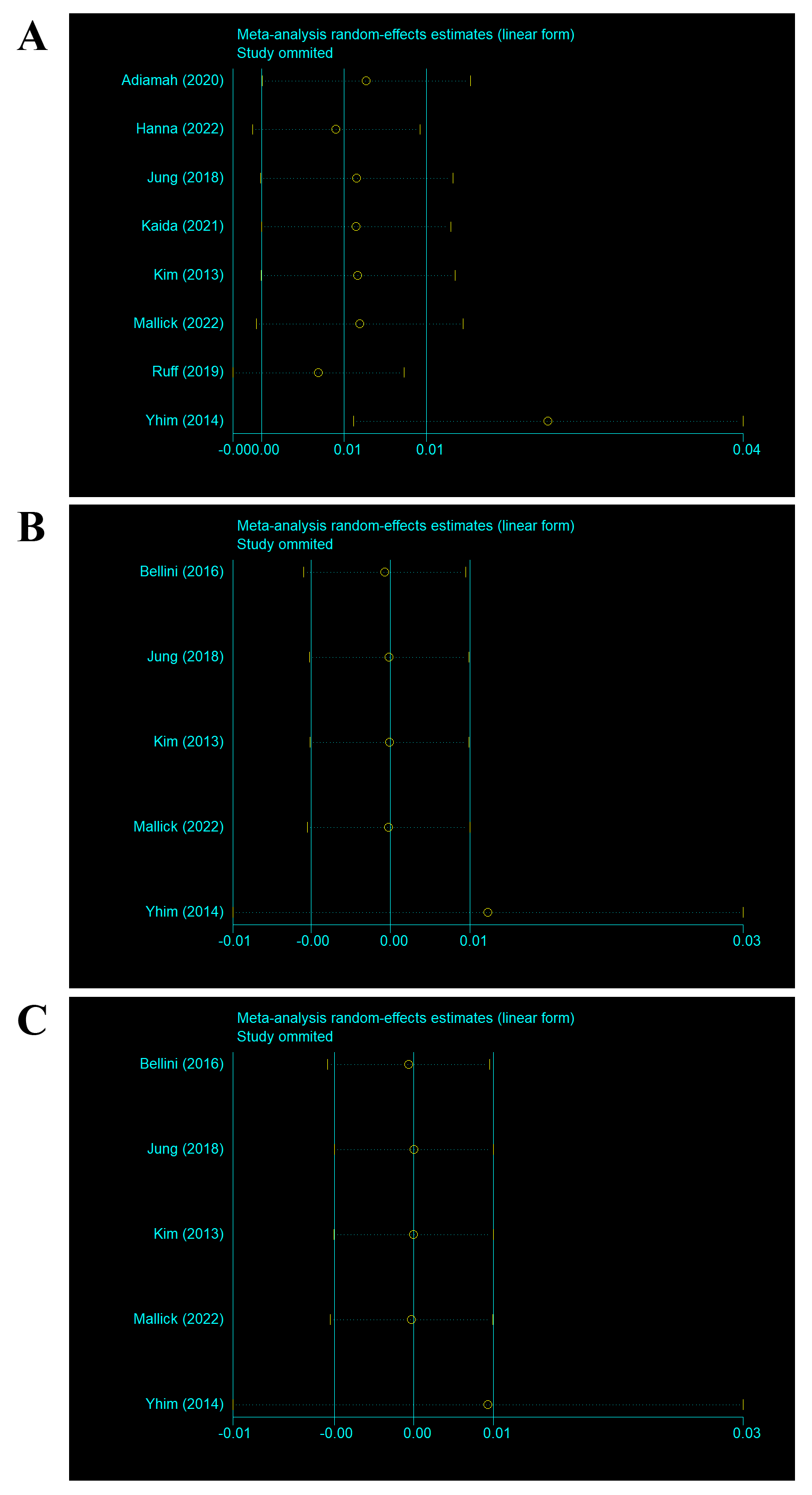
**

**Fig. S3 Forest plots of subgroup analysis for postoperative VTE within 1 month**

**
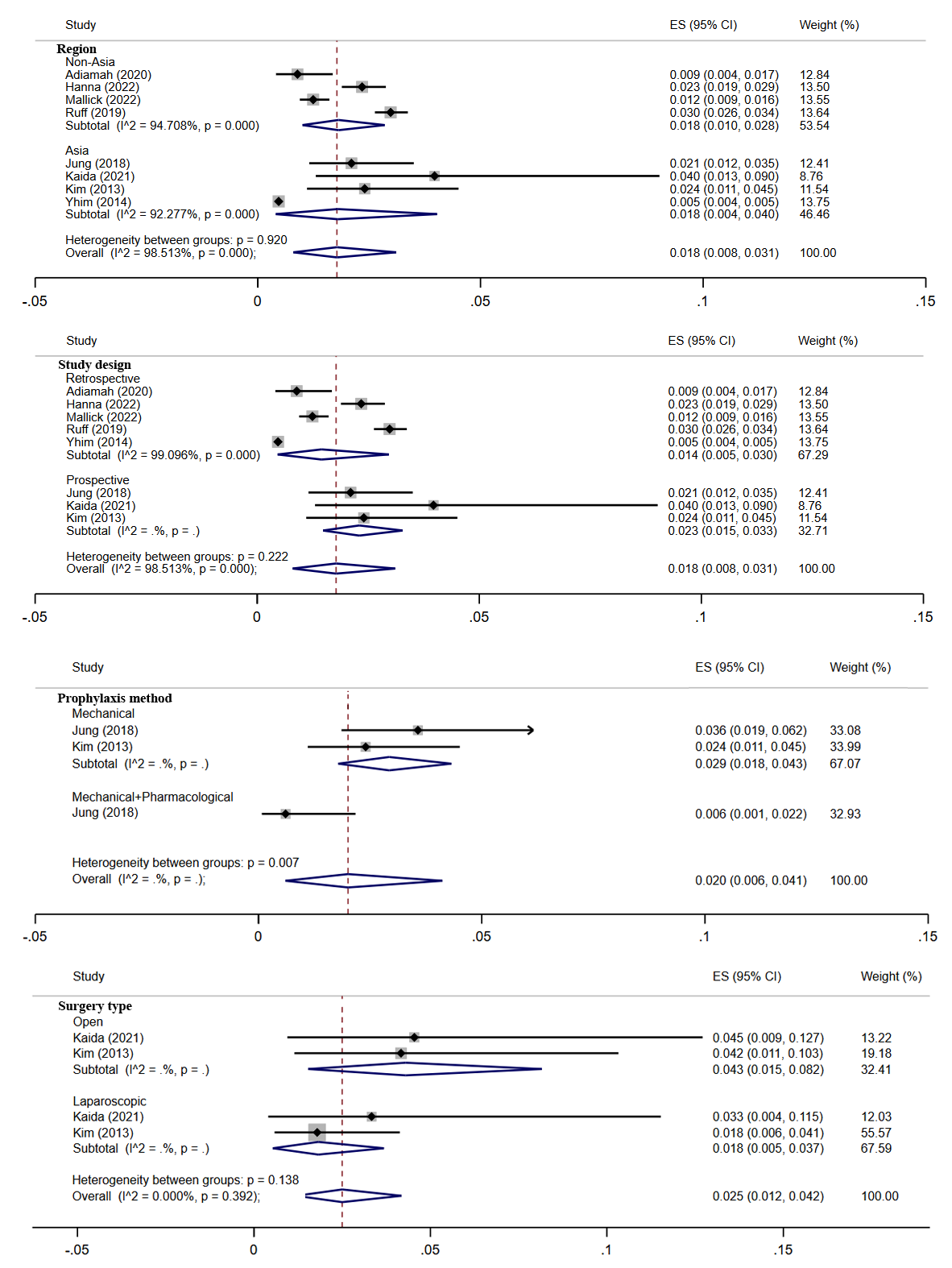
**

**Fig. S4 Forest plots of subgroup analysis for postoperative DVT within 1 month**

**
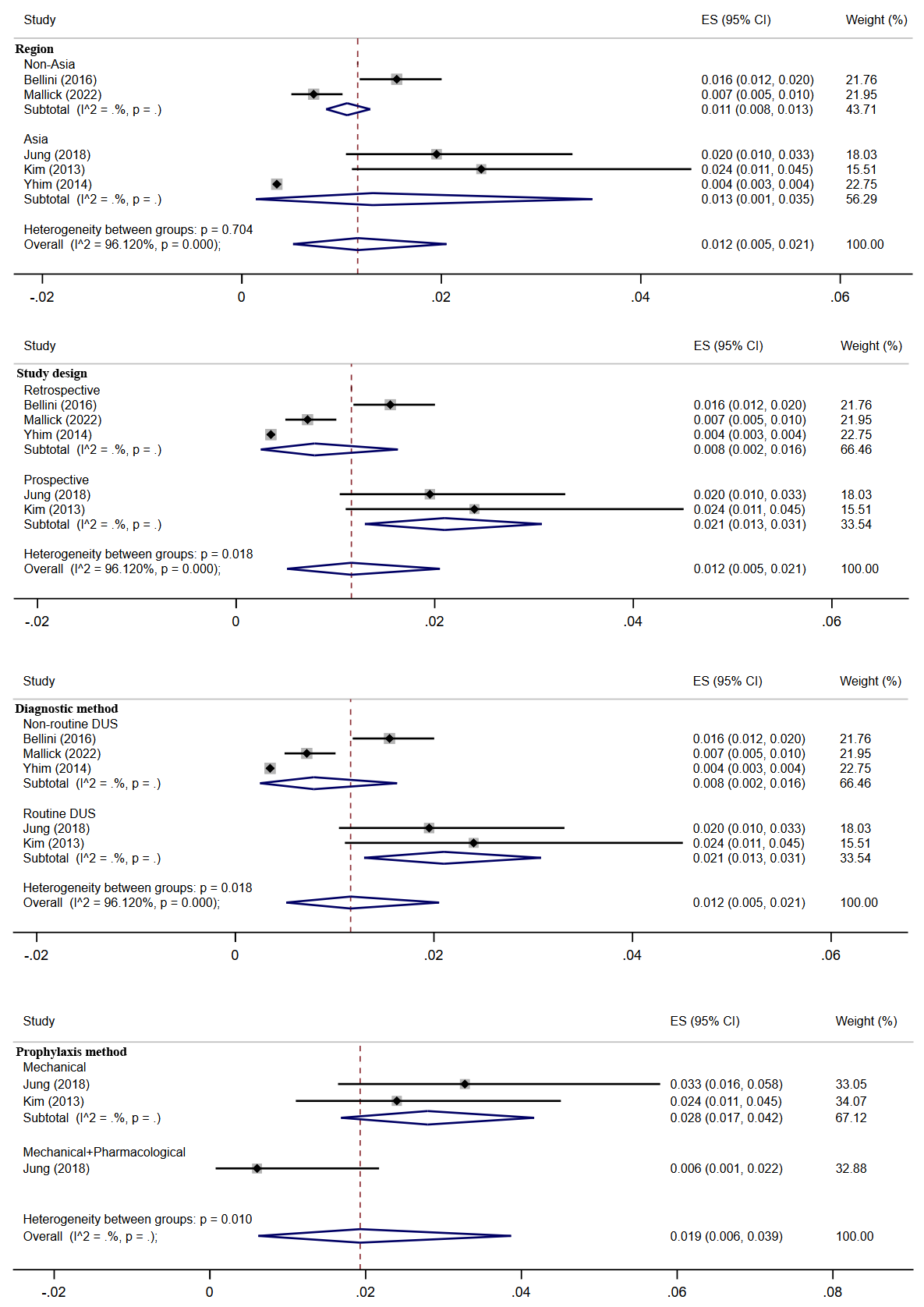
**

**Fig. S5 Forest plots of subgroup analysis for postoperative PE within 1 month**

**
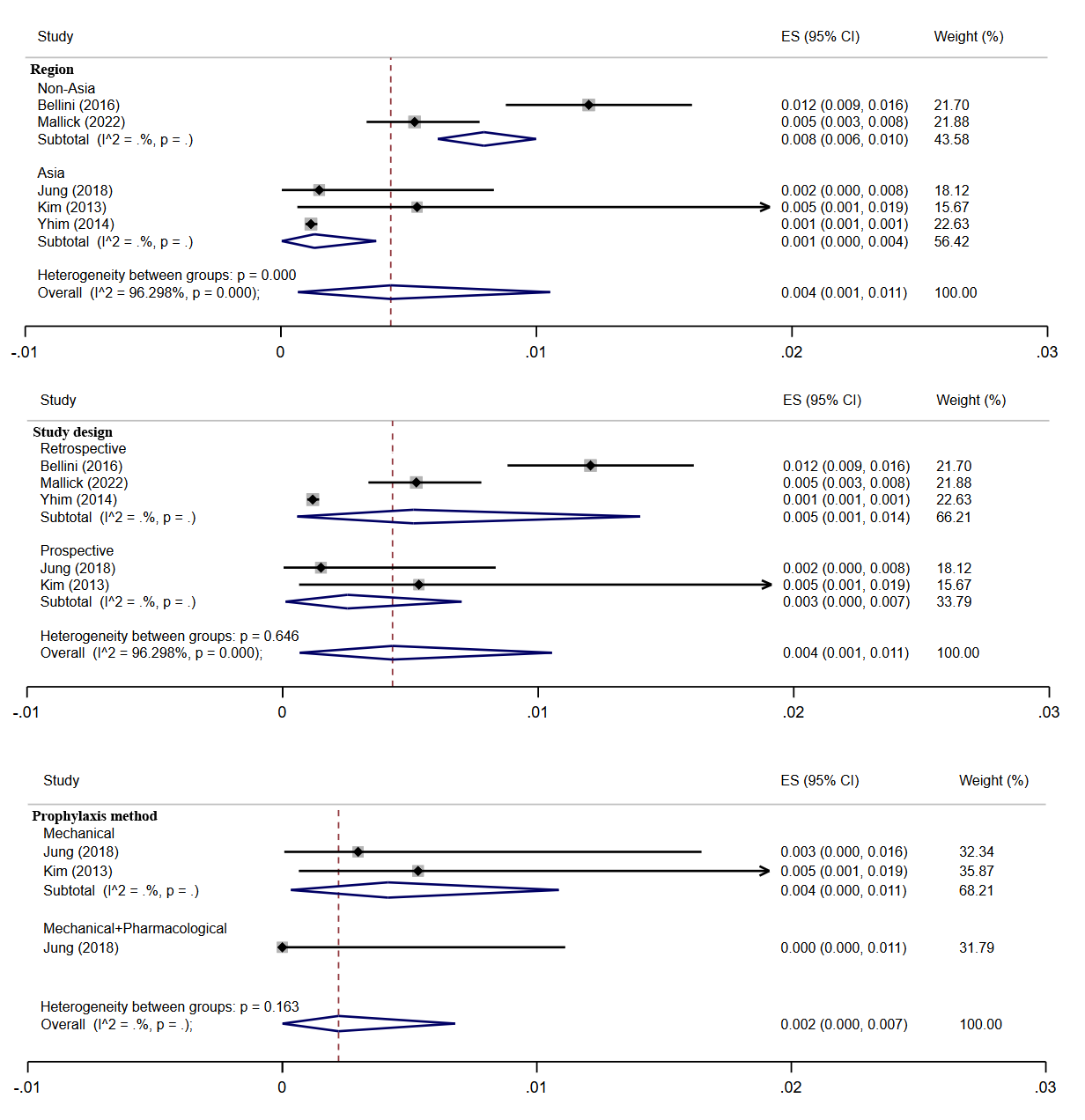
**

**Fig. S6 Funnel plots for the publication bias. (A) postoperative VTE within 1 month, (B) postoperative DVT within 1 month, (C) postoperative PE within 1 month**

**
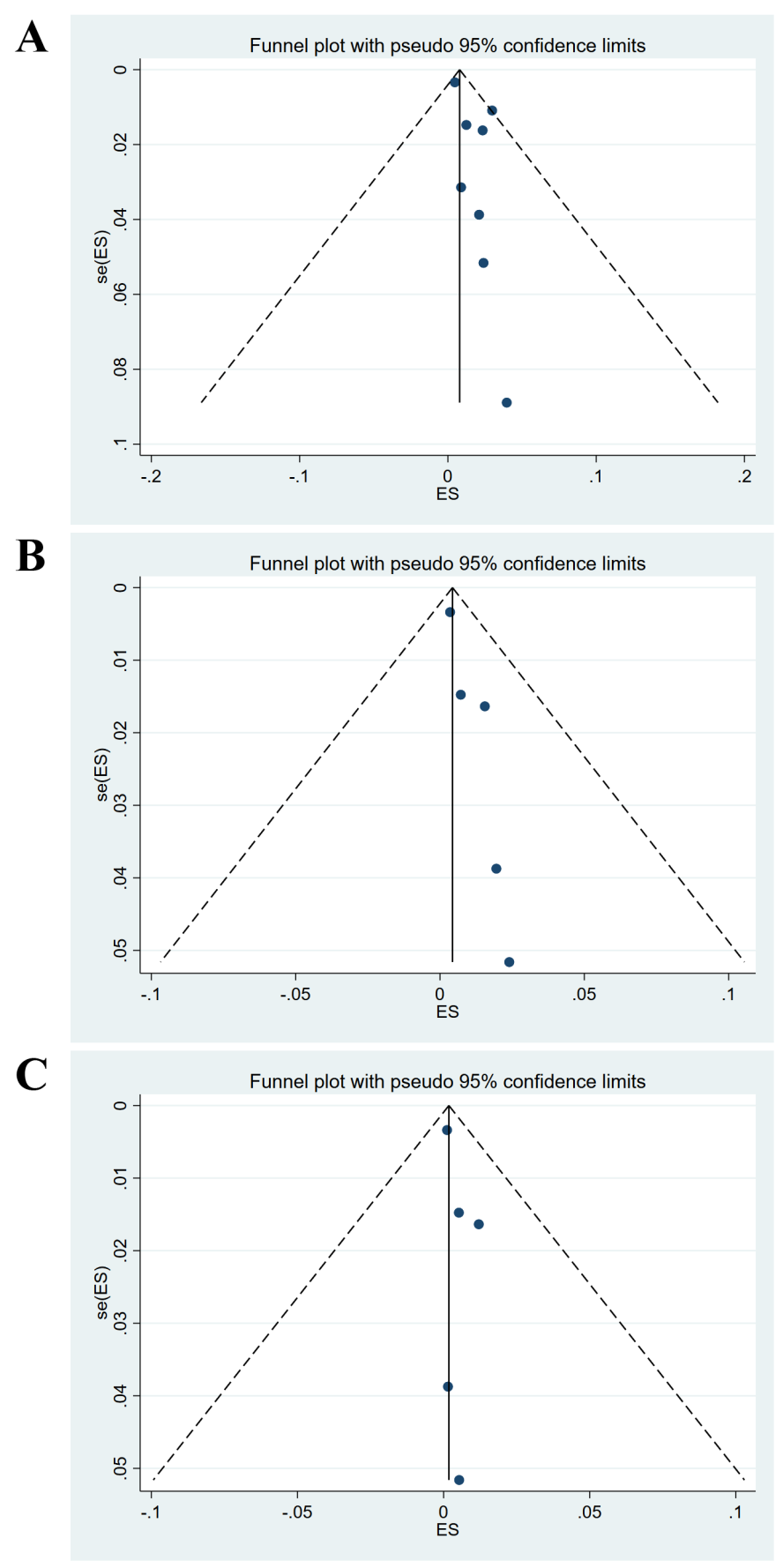
**

**Fig. S7 The trim-and-fill method for the publication bias of postoperative DVT within 1 month**

**
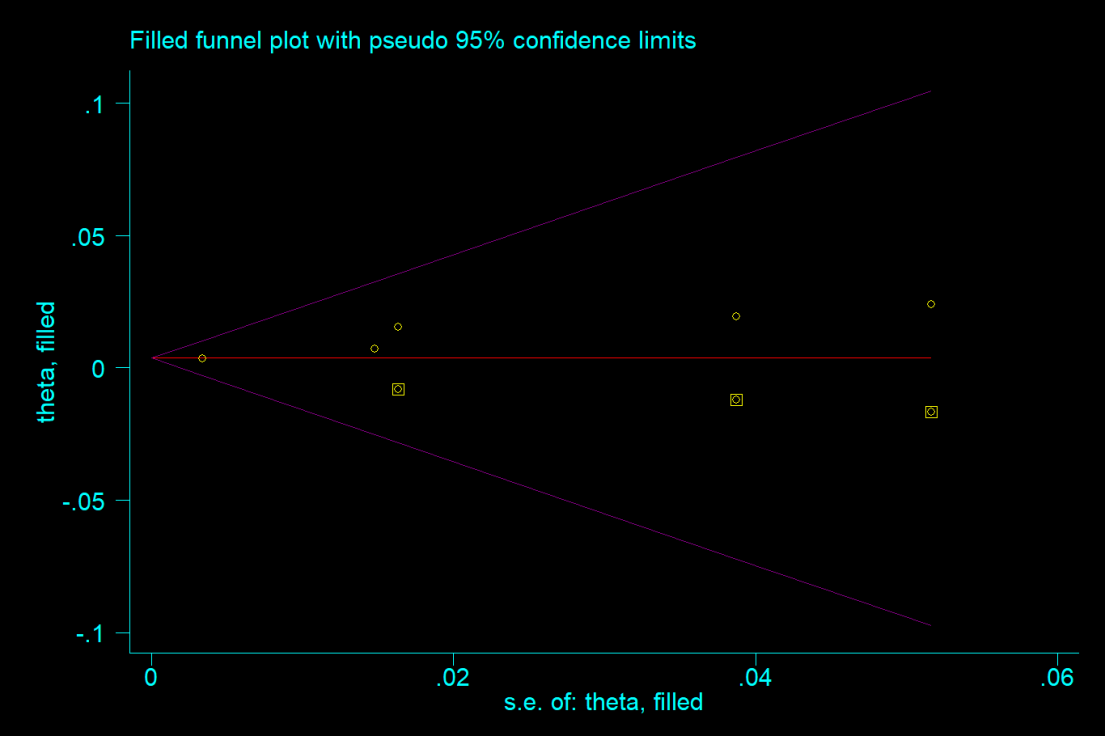
**

**
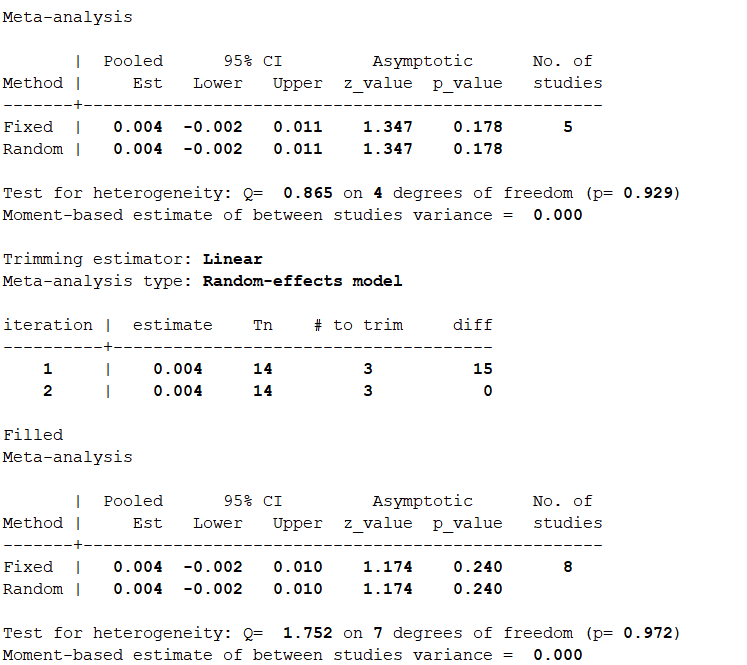
**
